# Expression of Hybrid Peptide EF-1 in *Pichia pastoris*, Its Purification, and Antimicrobial Characterization

**DOI:** 10.3390/molecules25235538

**Published:** 2020-11-26

**Authors:** Zhongxuan Li, Qiang Cheng, Henan Guo, Rijun Zhang, Dayong Si

**Affiliations:** State Key Laboratory of Animal Nutrition, Laboratory of Feed Biotechnology, College of Animal Science & Technology, China Agricultural University, Beijing 100193, China; Lee_zx20@yeah.net (Z.L.); chengqiangcool@163.com (Q.C.); ghn_657@cau.edu.cn (H.G.)

**Keywords:** heterologous expression, hybrid peptide, bacteriocin, antibacterial activity

## Abstract

EF-1 is a novel peptide derived from two bacteriocins, plantaricin E and plantaricin F. It has a strong antibacterial activity against *Escherichia coli* and with negligible hemolytic effect on red blood cells. However, the chemical synthesis of EF-1 is limited by its high cost. In this study, we established a heterologous expression of EF-1 in *Pichia pastoris*. The transgenic strain successfully expressed hybrid EF-1 peptide, which had a molecular weight of ~5 kDa as expected. The recombinant EF-1 was purified by Ni^2+^ affinity chromatography and reversed-phase high performance liquid chromatography (RP-HPLC), which achieved a yield of 32.65 mg/L with a purity of 94.9%. The purified EF-1 exhibited strong antimicrobial and bactericidal activities against both Gram-positive and -negative bacteria. Furthermore, propidium iodide staining and scanning electron microscopy revealed that EF-1 can directly induce cell membrane permeabilization of *E. coli*. Therefore, the hybrid EF-1 not only preserves the individual properties of the parent peptides, but also acquires the ability to disrupt Gram-negative bacterial membrane. Meanwhile, such an expression system can reduce both the time and cost for large-scale peptide production, which ensures its potential application at the industrial level.

## 1. Introduction

The need for new antibiotics that can be used for the treatment of human and livestock infections has been urgent due to the spread of multidrug-resistant pathogens. Antimicrobial peptides (AMPs) are considered a good starting point to develop of future antibiotics [1]. AMPs are typically naturally distributed amphipathic, cationic, small polypeptides [2] which secreted by various organisms such as vertebrates, plants, fungi, and bacteria [3]. They exhibit a great number of acting ways, for example, they can disrupt cell membrane through “barrel-stave” [4], “toroidal-pore” [5], and “carpet” [6] models and inhibit cell wall biosynthesis and cell division [7,8]. In addition, some of them can freely translocated across the bilayer and result in inhibition of DNA, RNA, and protein synthesis as well as the inhibition of cytosolic enzymatic activity [9,10,11]. AMPs have been proposed to have multiple targets, making them less likely to impose selective pressure on bacteria [9,12], which makes them considerable to be applied as novel antimicrobials for killing antibiotic-resistant bacterial pathogens [1]. Plantaricin EF (PlnEF), belonging to the type IIb bacteriocins, is produced by several *Lactobacillus plantarum* strains [13]. IIb bacteriocins are two-peptide bacteriocins and its antimicrobial activities rely on the actions of the two peptides [14]. PlnEF, consist of PlnE (33 amino acid residues) and PlnF (34 amino acid residues), is mainly antagonistic against Gram-positive bacteria [13] and fungi [15]. It is also active on Gram-negative bacteria when out membrane was pre-treatment with membrane destabilizing agents such as ethylene diamine tetraacetic acid (EDTA) [16]. According to previous studies, the helix–helix interaction between GxxxG motifs of PlnE and PlnF is essential for bacterial membrane permeabilization [13,17]. Therefore, the antimicrobial activity of PlnEF is majorly dependent on the synergistic actions of these two peptides [18].

The development of bacteriocins is limited by the difficulty in collecting them from natural producers [19,20], while the production of chemically synthetic peptides is costly. In contrast, recombinant heterologous expression of AMPs is a cost-effective method for antimicrobial peptide production. Heterologous expression can produce larger AMPs containing >30 amino acids, and this technologies for process development are mature. Recombinant expression used for therapeutic protein production can be used for AMPs production [21]. The methylotrophic *Pichia pastoris* expression system has been used extensively [22], which contains alcohol oxidase-1 (AOX-1) gene promoter that can be induced by methanol while repressed by glucose and glycerol. Such an expression system may be economical for small antimicrobial peptide (<10 kDa) production in a large scale [23,24,25].

In this study, to simplify the production of PlnEF and enhance its antimicrobial activity against Gram-negative bacteria, we established a heterologous expression of PlnEF in *P*. *pastoris* by hybridizing PlnE and PlnF. The antimicrobial activities of this hybrid peptide EF-1 were further examined against multiple bacterial strains.

## 2. Results

### 2.1. Construction of Expression Plasmids

The construction of expression plasmid is shown in Supplementary Materials Figure S1. To express the recombinant peptide with a native N-terminus, EF-1 gene fragment was cloned with the cleavage site of Kex2 at 5′ and 3′ ends, and the restrictive enzyme sites of *XhoI* and *XbaI* were also attached. Moreover, a His-tag at the C-terminus of the hybrid peptide EF-1 was added to facilitate peptide purification. The synthesized gene was cloned into pUC57 vector for double digestion with *XhoI* and *XbaI.* Then, the gene fragment was cloned into pPICZαA, at the downstream of alcohol oxidase gene (AOX1) promoter, to finalize the construction of the expression plasmid named pPICZαA-EF-1. The correct insertion of EF-1 gene fragment was confirmed by both PCR and direct nucleotide sequencing (data not shown).

### 2.2. Expression and Purification of Hybrid EF-1 Peptide

**Figure 1:**
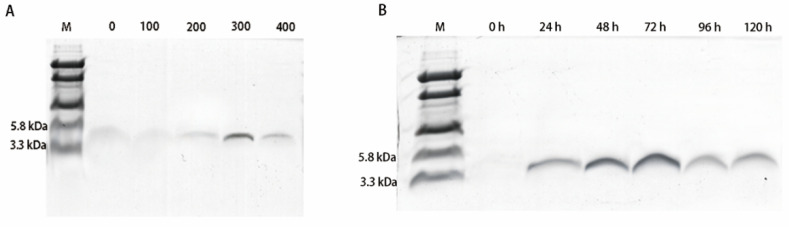
Tricine-SDS-PAGE of EF-1, (**A**) Tricine-SDS-PAGE of the purified secreted recombinant hybrid peptide EF-1 after 72 h methanol induction; lane M, mass weight markers; lane 2–6, purified EF-1 eluted with different concentrations of imidazole and in lane ~5 kDa (300 and 400 mM imidazole). (**B**) Tricine-SDS-PAGE of cell culture media after purified form expression; lane M, mass weight markers, lane 2–7, peptide expression after methanol (0–120 h) induction.

**Figure 2:**
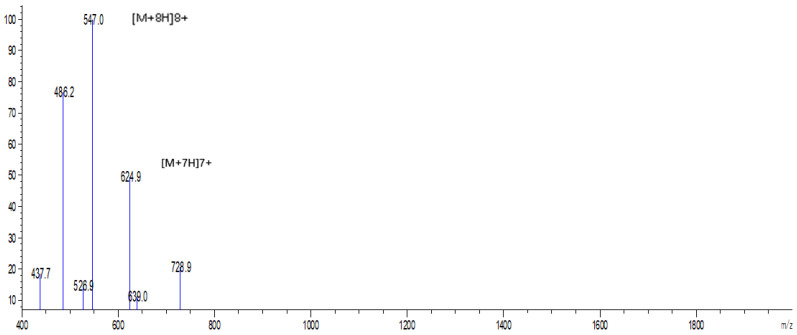
Electrospray ionization-mass spectrometry (ESI-MS) analysis of purified EF-1.

The expression plasmid pPICZαA-EF-1 was linearized by *SacI* digestion and transformed into *P. pastoris* X-33 by electroporation. A total of 117 Zeocin-resistant colonies were screened by PCR, and we confirmed that the target EF-1 gene sequence had been inserted into all of the positive transformants. The genetically engineered strains were induced by 0.5% pure methanol in Buffered Methanol-Complex Medium (BMMY) for five-day consecutive secretory EF-1 expression. The recombinant peptide could be detected after 24 h of induction, and the N-terminal α-factor secretion signal was removed from the recombinant EF-1 in cultural medium (Figure 1 and Figure 2). The eluted EF-1 had an expected molecular weight of ~5 kDa, while the final yield of EF-1 was ~32.65 mg/L with a purity of 94.9%.

### 2.3. Antimicrobial Activities of Synthesized PlnEF and Recombinant EF-1

**Figure 3:**
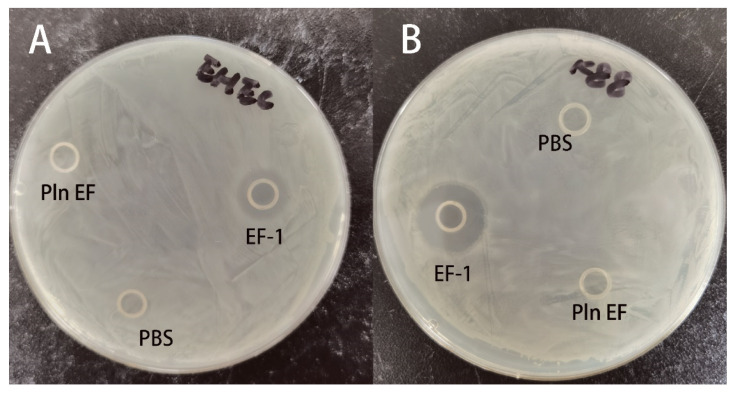
The inhibition zone of purified EF-1 (12.5 μM) and synthesized PlnEF (6.25 μM of PlnE and 6.25 μM of PlnF) against EHEC (**A**) and E. coli K88 (**B**) with sodium phosphate buffer as a negative control.

To determine and compare the antimicrobial activities of the recombinant EF-1 and synthesized PlnEF, we examined their minimum inhibitory concentrations (MICs), minimum bactericidal concentrations (MBCs), and inhibition zones (Table 1 and Figure 3). Both PlnEF and EF-1 exhibited strong antimicrobial activities against tested Gram-positive bacteria. The MICs of PlnEF against *S. aureus* and *M. luteus* were 25 and 12.5 μM, respectively, while those of EF-1 were 12.5 and 6.25 μM for both, respectively. However, PlnEF showed weak antibacterial activities against Gram-negative bacteria, with MICs higher than 100 μM against *S. flexneri*, *S.* Typhimurium, EHEC, and *E. coli* K88. In contrast, the MICs of purified EF-1 against *S. flexneri* and *S.* Typhimurium were both 25 μM. Moreover, the recombinant EF-1 also showed strong antimicrobial activities against EHEC (MIC = 6.25 μM) and *E. coli* K88 (MIC = 3.125 μM), with MICs at least 16-fold lower than those of PlnEF.

The recombinant EF-1 also exhibited strong bactericidal activities against Gram-negative bacteria. The MBCs of EF-1 against EHEC, *E. coli* K88, *S.* Typhimurium, and *S. flexneri* were 6.25, 3.125, 25, and 25 μM, which were at least 16-, 32-, 4-, and 8-fold lower than those of synthesized PlnEF, respectively. For Gram-positive bacteria, both EF-1 and PlnEF exhibited the same bactericidal activities (MBC = 12.5 μM) against *M. luteus*, while the MBC of EF-1 against *S. aureus* (12.5 μM) was 4-fold lower than that of PlnEF (50 μM). These results indicated that compared with PlnEF, EF-1 displayed overall better antimicrobial and bactericidal activities.

In addition, the inhibition zones of the two peptides against EHEC and *E. coli* K88 (Figure 3A, B) were measured by disk diffusion method. The results evidently illustrated that the recombinant hybrid EF-1 substantially inhibited the growth of EHEC and *E. coli* K88. Besides, the inhibition zones were consistent with MICs and MBCs which indicated that *E. coli* K88 was more sensitive to EF-1.

### 2.4. EF-1 Induces Bacterial Membrane Permeabilization

**Figure 4:**
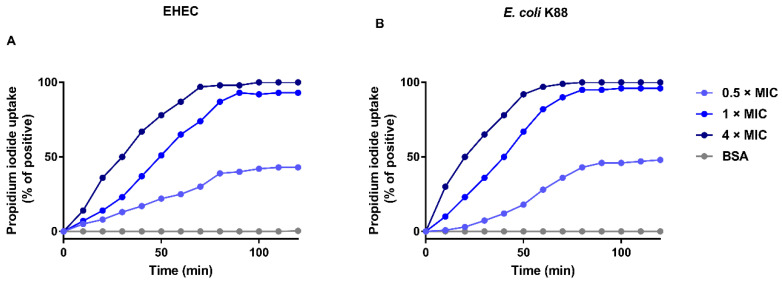
Propidium iodide uptake by enterohemorrhagic EHEC (**A**) and *E. coli* K88 (**B**) in the presence of purified recombinant EF-1, 0.05% (*w*/*v*) SDS the maximum fluorescence, BSA as a negative control. (**A**) 0.5, 1 and 4 × MIC of purified EF-1 against EHEC were the concentration of 3.125, 6.25, and 25 μM. (**B**) 0.5, 1, and 4 × MIC of purified EF-1 against *E. coli* K88 were the concentration of 1.5, 3.125, and 6.25 μM.

To investigate the effect of EF-1 on bacterial membrane, we measured the PI uptake by EHEC (Figure 4A) and *E. coli* K88 (Figure 4B) under various concentrations of EF-1 treatments. We found that EF-1 increased PI uptake by EHEC and *E. coli* K88 in a dose-dependent manner. To be specific, at 0.5 × MIC, EF-1 treatment resulted in 43% and 48% PI uptake after 120 min for EHEC and *E. coli* K88, respectively. The PI uptake by EHEC treated with EF-1 for 60 min (1 × MIC) and 30 min (4 × MIC) was increased by 50%, and it was increased by 90% after 90 min (1 × MIC) and 70 min (4 × MIC) of treatment, respectively. In contrast, 50% more PI uptake by *E. coli* K88 was achieved when the bacteria were treated with EF-1 for 40 and 20 min at 1 × and 4 × MICs, respectively, while at the same concentrations treatment after 80 and 50 min, 90% more PI uptake was achieved. These findings indicated that the bactericidal activity of EF-1 was through inducing bacterial membrane permeabilization and it exhibit a dose-dependent manner.

**Figure 5:**
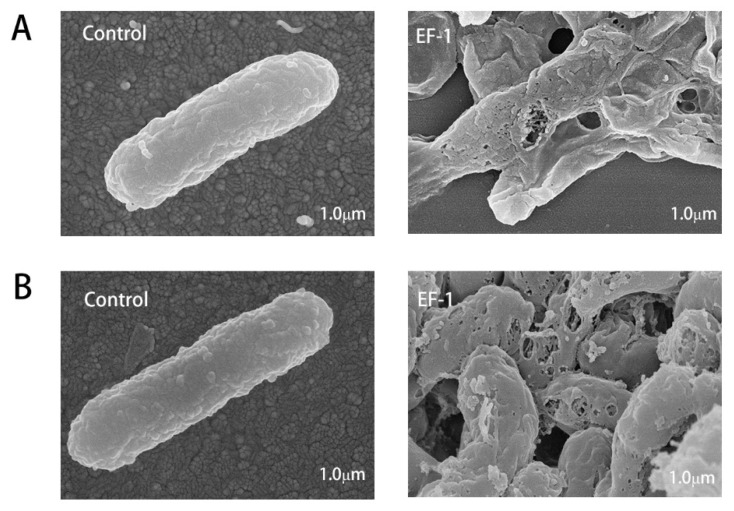
Scanning electron microscopy of EHEC (**A**) and *E. coli* K88 (**B**) cells treated with hybrid EF-1 for 2 h. Scale bars: 1.0 μm.

Furthermore, scanning electron microscopy was performed to visualize morphological changes of EHEC and *E. coli* K88 that were exposed to EF-1 (1 × MIC) for 2 h (Figure 5). The images showed that EF-1 created pores on the bacterial cells, which compromised the integrity of bacterial cell membrane, and confirmed the abovementioned mechanism of action through which EF-1 kills Gram-negative bacteria.

### 2.5. Hemolytic Activity of Purified Hybrid EF-1

**Figure 6:**
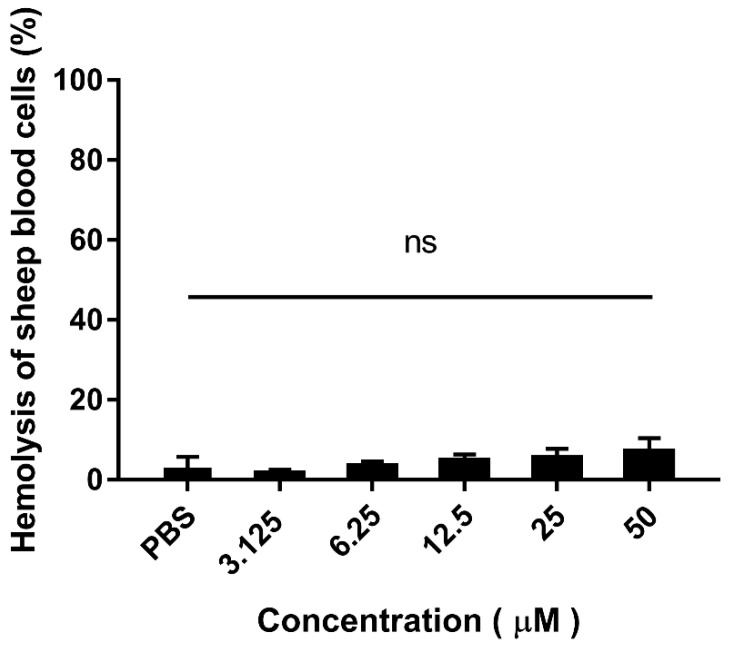
Hemolytic effect of purified hybrid peptide EF-1 in contradiction of sheep RBCs. The data resemble the mean ± standard deviation; ns—not significant.

Additionally, the hemolytic activity of purified recombinant EF-1 to sheep erythrocyte cells shown in Figure 6. At the concentration of 50 μM of EF-1, the hemolysis of EF-1 against sheep blood cells is less than 10%. The hybrid peptide treated with red blood cells (RBCs) perceived no significant hemolytic activity as compared with the PBS group. These outcomes provide evidence that hybrid EF-1 doesn’t have hemolytic properties.

## 3. Discussion

A growing number of natural AMPs have been shown to be potentially applied for treating pathogenic infections. AMPs can be manufactured through different approaches, such as extraction from natural sources, solid-phase synthesis, and genetic engineering [19,20]. However, the low efficiency of extraction from nature producers and the high cost of chemical synthesis limit the large-scale production of AMPs. In contrast, recombinant expression of AMPs (e.g., in *E. coli* expression system) has been widely applied, but the expression is usually compromised by their protease sensitivity and lethality to the microbial producers [26].

Although a variety of fusion protein techniques have been developed to overcome the abovementioned limitations [27,28], they lead to a low expression of target peptides (10–30 mg/L of fusion proteins and 1–5 mg/L peptides) [29,30]. Besides, an additional step of fusion tag removal by exogenous enzymes is also required for downstream purification, which is both time- and cost-consuming. We selected *P. pastoris* for hybrid EF-1 expression, because these host cells have the advantage of being resistance to AMP-mediated killing [21]. The expression vector pPICZαA contains an AOX1 promoter and an α-factor secretion signal, which can ensure a stable expression and secretion of heterologous peptides [12]. In the present study, the hybrid peptide EF-1 was successfully expressed in *P. pastoris* through pPICZαA transformation. Moreover, downstream purification of EF-1, facilitated by the His-tag, was efficiently achieved by Ni^2+^ affinity chromatography and RP-HPLC. The yield of purified EF-1 (~32.65 mg/L) is close to those of CA-MA [31] and cecropin AD [25] described previously.

In recent years, novel peptides have been designed by hybridizing different AMPs to improve their individual properties [32]. The replacement of conserved amino acid residues does not affect their activity, and several suitable sequences can enhance the efficiency of hybrid peptides [3]. PlnEF, a cationic bacteriocin, has been demonstrated to counteract Gram-positive bacteria through disrupting bacterial membrane. The permeabilization ability of PlnEF is dependent on the helix–helix interaction between GxxxG motifs [33,34]. Moreover, both the N-terminal aromatic amino acid residues of PlnE and the C-terminal of PlnF play critical roles in their antimicrobial properties by positioning in the interface of bacterial membrane, and thus anchoring the peptides on the membrane [35]. Besides, cationic bacteriocins can also interact with anionic bacterial membrane by electrostatic forces [35]. In this study, we hybridized two peptides, forming the novel EF-1 to reduce the cost of PlnEF production. The designed peptide not only reserves the aromatic amino acid residues and the GxxxG motifs of PlnEF, but also possesses an increased net charge (PlnE: +5; PlnF: +3; EF-1: +7) and improved amphipathicity and hydrophobicity. Therefore, its electrostatic interaction with bacterial membrane is intensively enhanced [35,36]. Our antimicrobial activity results demonstrated EF-1 have antagonistic effect on Gram-positive bacteria, also, the novel peptide exhibited bactericidal activities against Gram-negative bacteria. For Gram-negative bacteria, PlnEF displayed antimicrobial activity only when their out membrane was pre-treatment by destabilization agents [16]. However, in the present study, hybrid peptide EF-1 showed antibacterial activity to Gram-negative bacteria without the agents’ pre-treatment, which indicated that the novel peptide has stronger antibacterial activity than PlnEF for Gram-negative bacteria. Among all the tested bacteria, novel peptide EF-1 had the relative lower MICs and MBCs against EHEC and *E. coli* K88 than against other bacteria, suggesting its higher *E. coli*-specificity. However, the underlying mechanism of this specificity requires further studies.

To further understand the antimicrobial activity of hybrid EF-1, PI uptake assay was measured to reflect the effect of EF-1 on bacterial membrane integrity. The results demonstrated EF-1 inducing EHEC and *E. coli* K88 membrane permeabilize, and this effect was concentration dependent. As PI uptake increased when more EF-1 was added at the same incubation time. The activity was further confirmed by SEM. As observed by SEM, EF-1 created pores on EHEC and *E. coli* K88 cells, which compromised the integrity of bacterial cell membrane. These results proved the novel hybrid EF-1 kills Gram-negative bacteria through inducing bacterial membrane permeabilization. Finally, we tested the hemolysis of recombinant peptide on sheep RBCs. There is no significant Hemolytic activity of EF-1 compared with PBS, which means EF-1 have application potential in internal medicine.

## 4. Materials and Methods

### 4.1. Regents and Enzymes

The restriction enzymes *XhoI, XbaI* and *SacI* were purchased from NEB (New England Biotech, Hitchin, UK). Zeocin, pPICZαA, *P. pastoris* X-33 and low range protein marker were from Thermofisher (Waltham, MA, USA). The *FastPfu* Fly DNA polymerase and DNA marker were from TransGen (Beijing, China).

### 4.2. Bacterial Strains, Plasmid

Indicator strains *Staphylococcus aureus* CVCC 1882, *Micrococcus luteus* CMCC 28001 *Salmonella* Typhimurium ATCC 14028, *Shigella flexneri* CMCC 51572, Enterohemorrhagic *Escherichia coli* (EHEC) O157:H7 and Enterotoxigenic *Escherichia coli* K88 were saved in our lab.

The *Picha pastoris* X 33 and plasmid pPICZαA were purchased from Invitrogen (Thermofisher, Waltham, MA, USA). *E. coli* DH5α chemically competent cells were from TransGen (Beijing, China).

### 4.3. Chemical Synthesis of Plantaricin EF

The two peptides Plantaricin EF were synthesized by 9-fluorenylmethoxycarbony solid-phase synthesis chemistry and purified by a reverse-phase semi-preparative HLPC.

### 4.4. Construction of Expression Vectors

The preferred codons of *P. pastoris* based on the hybrid EF-1 peptide amino acid sequences with 6 × His tag were optimized by JAVA codon adaption tool (JCAT, http://www.jcat.de/Start.jsp). Sequence encoding EF-1 which containing *XhoI*, *XbaI* and introduced in-frame of the α-factor secretion signal in pPICZα-A expression vector was synthesized by Genewiz (Suzhou, China) and cloned into pUC57. The gene of EF-1 was amplified using primers (P1: 5′-CCGCTCGAGAAAAGAGAGGCTGAAGC-3′; P2: 5′-TGCTCTAGATTAATGGTGGTGATGGTGATGT-3′) and PCR (94 °C for 4 min, 94 °C for 30 s, 55 °C for 30 s, 72 °C for 40 s, 30 cycles) and finally extension for 7 min at 72 °C. The PCR products were confirmed by 1.5% gel electrophoresis and purified by DNA gel extraction kit (Omega, Norwalk, CT, USA). The purified PCR products then digestion by *XhoI, XbaI* and inserted to pPICZαA using T4 DNA Ligase (NEB). The ligation mixture was transformed into *E. coli* DH5α competent cells, positive clones were selected by low salt Luria-Bertani agar (1% tryptone, 0.5% yeast extract, 0.5% NaCl, 1.8% agar powder) with 25 μg/mL Zeocin. Plasmid DNA was isolated by TIANperp Midi Plasmid Kit (Tiangen, Beijing, China) and then verified by PCR amplification and sequencing.

### 4.5. Expression of EF-1 Peptide in P. pastoris

The constructed pPICZαA-EF-1 plasmid was linearized by *SacI* and transformed into *P. pastoris* X-33 cells by electroporation manufacturer’s instructions. An alone pPICZαA vector, as a negative control, was also inserted into pPICZαA X-33. After transformation, Zeocin-resistant colonies were allowed to grow in YPDS agar (1% yeast extract, 2% peptone, 2% dextrose, 1 M sorbitol, 1.8% agar) with 100 μg/mL Zeocin. Genomic DNA of positive colonies was isolated using a TIANamp Yest DNA Kit (Tiangen, Beijing, China). Target gene was amplified with following program: 94 °C for 5 min, denaturing at 94 °C for 45 s, annealing at 56 °C for 30 s, extension at 72 °C for 45 s, 28 cycles and finally extension for 10 min at 72 °C. The PCR products then separated by 1.5% gel electrophoresis, purified with DNA gel extraction kit (Omega), and sequencing by Genewiz (Suzhou, China).

The EF-1 hybrid peptide was expressed in the condition which was optimized follow by manufacturer’s instructions (0.5% methanol *v*/*v*, pH 6.0, 28 °C) in Buffered Methanol-Complex Medium (BMMY, 1% yeast extract, 2% peptone, 100 mM potassium phosphate buffer, pH 6.0, 1.34% YNB, 4 × 10^−5^% biotin, and methanol). The positive cells were cultured for about 18 h in a shaking flask comprising Buffered Glycerol-Complex Medium (BMGY, 1% yeast extract, 2% peptone, 100 mM potassium phosphate buffer, pH 6.0, 1.34% YNB, 4 × 10^−5^% biotin, and 1% glycerol) to OD_600_ = 2.0. Cells were harvested under 5000× *g*, 5 min at room temperature and resuspended by fresh BMMY to OD_600_ = 1.0 to induce expression of EF-1. In 120-h methanol induction, 1-mL expression supernatant was harvested every 24 h.

### 4.6. Purification of Recombinant EF-1 Peptide

Recombinant hybrid peptide was purified by Ni-NTA Sepharose column (GE Healthcare, Chicago, IL, USA). The expressed culture medium centrifuged at 10,000× *g* for 15 min, 4 °C, and the supernatant was collected and then filtrated by 0.22 μm filter (Millipore, Burlington, MA, USA). Ni-NTA Sepharose column was pre-equilibrated with buffer containing 20 mM Tris-HCl, 300 mM NaCl, 40 mM imidazole, pH 7.5. The supernatants were loaded onto Ni-NTA column and washed with equilibration buffer. The bound peptide then eluted by buffer (20 mM Tris-HCl, 300 mM NaCl, 40 mM imidazole, pH 7.5) five times. Fractions were pooled and then concentrated by Amicon Ultra centrifugal filters (Millipore, Burlington, MA, USA). The collected peptide was further purified by reversed-phase high performance liquid chromatography (RP-HPLC). Purified peptide was quantified through Bradford assay kit (Sangon Biotech, Shanghai, China) using bovine serum albumin as a standard and analyzed by 16%/6 M urea Tricine-sodium dodecyl sulfate-polyacrylamide gel electrophoresis (Tricine-SDS-PAGE) [37].

### 4.7. Analysis of EF-1 by Electrospray Ionization Mass

The recombinant peptides were analyzed by electrospray ionization-mass spectrometry (ESI-MS) using an instrument SHIMADZU LCMS-2020 (SHIMADZU, Tokyo, Japan). The peptides were diluted using Milli-Q water with solution of α-cyano-4-hydroxycinnamic acid containing 50% (*v*/*v*) acetonitrile and 0.1% (*v*/*v*) TFA. The spectra were acquired over a mass/charge (*m*/*z*) range of 400-2000 in direct and reflective mode.

### 4.8. Antimicrobial Activity

#### 4.8.1. Inhibition Zone

Disk diffusion method of peptides with the *E. coli* K88 and EHEC as indicator bacteria were measured. The indicator strains were grown in Mueller–Hinton Broth (MH, Solarbio, Beijing, China) at 37 °C, 200 rpm overnight. Harvested growth culture was diluted to 10^7^ CFU/mL and then 200 μL diluted solution was spread on MH plates. Oxford cups were placed on the agar, and 100 μL of purified peptide and synthesis plantaricin EF were added to these cylinders. The same volume of PBS was added as a negative control. The inhibition zone was measured after incubation at 37 °C over 12 h.

#### 4.8.2. Minimal Inhibitory Concentrations and Minimum Bactericidal Concentrations

The minimal inhibitory concentration (MIC) and minimum bactericidal concentrations (MBC) were measured as previously described [38]. The indicator strains were cultured at 37 °C overnight and diluted to the concentration of 10^6^ CFU/mL using fresh MH broth. Recombinant peptides were serially diluted in fresh MH broth. Then, 50 μL of peptide solution and 50 μL of diluted bacteria growth culture were dispensed into a round bottom microtiter plate (Corning, Corning, NY, USA). The bacteria in the final desired inoculum were 5 × 10^5^ CFU/mL each well. A blank control containing 100 μL fresh MH broth, and a growth control containing 50 μL diluted bacteria culture with 50 μL MH broth were set. The plate was incubated at 37 °C for 20 h. MIC was calculated as the lowest concentration of the antimicrobial agent that inhibits visible growth of indicator strains. Then 20 μL of mixture from each well was collected and spread on MH agar plates to detection MBC. The agar plates were incubated at 37 °C overnight and MBC was calculated as the lowest peptide concentration killing 99% of the bacteria inoculum. Results were based on three independent experiments performed in duplicate.

#### 4.8.3. Membrane Integrity Evaluation

Propidium iodide (PI) uptake assay was used to evaluate the bacterial membrane integrity. PI cannot cross the intact bacterial envelope, therefore, the PI uptake indicates the extent of bacteria membrane damage. Overnight growth bacteria culture was diluted to 5 × 10^5^ CFU/mL in MH broth containing 5.5 μg/mL PI (Thermo Fisher, Waltham, MA, USA). Bacterial samples were added to a black 96-well plate (Corning, Corning, NY, USA) and placed into the Spectramax plate reader (Synergy 4, BioTek, Winooski, VT, USA) that was preequilibrated to 37 °C. Before adding peptides, an initial reading (T_0_, 0 s) was measured, and then incubated with recombinant EF-1 and synthesis plantaricin EF for 2 h. During continued 2-h reaction, fluorescence readings (excitation, 535 nm; emission, 617 nm) were recorded every 10 min. A negative control, BSA, and a positive control, 0.05% (*w*/*v*) SDS was included. Membrane permeabilization activity was measured against the maximum fluorescence from positive control and the T_0_ readings were subtracted from each time from each well for data analysis.

#### 4.8.4. Scanning Electron Microscopy

Bacterial growth culture was collected and incubated with 1 × MIC peptide for 2 h at 37 °C. Bacteria were centrifuged for 5 min at 5000× *g*, washed in PBS three times. Bacterial samples were resuspended in 2.5% glutaraldehyde and incubated overnight at 4 °C. After dehydrated by gradient alcohol solutions, samples were freeze-dried and coated with gold and imaged by Quanta200 (FEI).

### 4.9. Hemolytic Assay

Concentrations of peptide that caused 50% hemolysis on mouse red blood cells (RBCs) at 414 nm were measured to evaluate the hemolytic activities [39]. Four milliliters of fresh mouse RBCs were centrifuged at 800× *g* for 10 min and washed three times with PBS. Peptides were dissolved in PBS with various concentrations and incubated for 1 h at 37 °C. The sample was centrifuged at 800× *g* for 10 min and absorbance (Abs) of the supernatant was measured at 414 nm. The PBS and 0.1% (*v*/*v*) Triton X-100 were used as the negative/positive control.

$$\%\;\mathrm{hemolysis}=\frac{\mathrm{Abs}\;414\;\mathrm{of}\;\mathrm{sample}\;-\;\mathrm{Abs}\;414\;\mathrm{of}\;\mathrm{negative}\;\mathrm{control}\;\left(\mathrm{PBS}\right)\times 100}{\mathrm{Abs}\;414\;\mathrm{of}\;\mathrm{positive}\;\mathrm{control}\;(\mathrm{Triton}\;X-100)}$$

### 4.10. Statistical Analyses

Data were presented as mean ± SD. For statistical analysis, one-way analysis of variance (ANOVA) and Duncan’s multiple range tests were used and carried out with SPSS 19.0 (SPSS Inc, Chicago, IL, USA). Differences with a *p* < 0.05 and *p* < 0.01 were considered statistically significant.

## 5. Conclusions

In summary, an efficient system has been developed to express and produce a hybrid peptide EF-1 in *P. pastoris*. The recombinant EF-1 exhibits strong bactericidal activities against EHEC and *E. coli* K88 by compromising their cell membrane integrity. Additionally, EF-1 has no hemolytic activity on red blood cells. This study delivers a promising strategy for expressing and purifying hybrid antibacterial peptide EF-1, which might facilitate the development of novel antimicrobials.

## Figures and Tables

**Table 1:** Minimum inhibitory concentrations (MICs) and minimum bactericidal concentrations (MBCs) of synthesized Plantaricin EF (PlnEF) and recombinant EF-1 against indicator bacterial strains.

Indicator Strains	MIC (μM)		MBC (μM)	
	Synthesized PlnEF	Recombinant EF-1	Synthesized PlnEF	Recombinant EF-1
*S. aureus* CVCC 1882	25	12.5	50	12.5
*M. luteus*CMCC 28001	12.5	6.25	12.5	12.5
*S.* Typhimurium ATCC 14028	>100	25	>100	25
*S. flexneri*CMCC 51572	>100	25	>100	25
EHEC	>100	6.25	>100	6.25
*E. coli* K88	>100	3.125	>100	3.125

*S. aureus*—*Staphylococcus aureus*; *M. luteus*—*Micrococcus luteus*; *S.* Typhimurium—*Salmonella* Typhimurium; *S. flexneri*—*Shigella flexneri*; EHEC—Enterohemorrhagic *Escherichia coli* O157:H7; *E. coli* K88—Enterotoxigenic *Escherichia coli* K88.

## Supplementary Materials

The following are available online, Figure S1: The map of expression vector construction.

## References

[1] Kang S.J., Park S.J., Mishig-Ochir T., Lee B.-J. (2014). Antimicrobial peptides: Therapeutic potentials. Expert Rev. Anti Infect. Ther..

[2] Hwang P.M., Vogel H.J. (1998). Structure-function relationships of antimicrobial peptides. Biochem. Cell Biol..

[3] Shah Y., Sehgal D., Valadi J.K. (2017). Recent trends in antimicrobial peptide prediction using machine learning techniques. Bioinformation.

[4] Yang L., Harroun T.A., Weiss T.M., Ding L., Huang H.W. (2001). Barrel-stave model or toroidal model? A case study on melittin pores. Biophys. J..

[5] Sengupta D., Leontiadou H., Mark A.E., Marrink S.-J. (2008). Toroidal pores formed by antimicrobial peptides show significant disorder. Biochim. Biophys. Acta.

[6] Oren Z., Shai Y. (1997). Selective Lysis of Bacteria but Not Mammalian Cells by Diastereomers of Melittin:  Structure—Function Study. Biochemistry.

[7] Martin N.I., Breukink E. (2007). Expanding role of lipid II as a target for lantibiotics. Future Microbiol..

[8] El Jastimi R., Edwards K., Lafleur M. (1999). Characterization of Permeability and Morphological Perturbations Induced by Nisin on Phosphatidylcholine Membranes. Biophys. J..

[9] Brogden K.A. (2005). Antimicrobial peptides: Pore formers or metabolic inhibitors in bacteria?. Nat. Rev. Microbiol..

[10] Fjell C.D., Hiss J.A., Hancock R.E.W., Schneider G. (2011). Designing antimicrobial peptides: Form follows function. Nat. Rev. Drug Discov..

[11] Guilhelmelli F., Vilela N., Albuquerque P., Derengowski L.D.S., Silva-Pereira I., Kyaw C.M. (2013). Antibiotic development challenges: The various mechanisms of action of antimicrobial peptides and of bacterial resistance. Front. Microbiol..

[12] Friedrich C.L., Moyles D., Beveridge T.J., Hancock R.E.W. (2000). Antibacterial action of structurally diverse cationic peptides on gram-positive bacteria. Antimicrob. Agents Chemother..

[13] Fimland N., Rogne P., Fimland G., Nissen-Meyer J., Kristiansen P.E. (2008). Three-dimensional structure of the two peptides that constitute the two-peptide bacteriocin plantaricin EF. BBA Proteins Proteom..

[14] Garneau S., Martin N.I., Vederas J.C. (2002). Two-peptide bacteriocins produced by lactic acid bacteria. Biochimie.

[15] Sharma A., Srivastava S. (2014). Anti-Candida activity of two-peptide bacteriocins, plantaricins (Pln E/F and J/K) and their mode of action. Fungal Biol..

[16] Pal G., Srivastava S. (2014). Inhibitory effect of plantaricin peptides (Pln E/F and J/K) against *Escherichia coli*. World J. Microbiol. Biotechnol..

[17] Hauge H.H., Mantzilas D., Eijsink V.G.H., Nissen-Meyer J. (1999). Membrane-Mimicking Entities Induce Structuring of the Two-Peptide Bacteriocins Plantaricin E/F and Plantaricin J/K. J. Bacteriol..

[18] Nissen-Meyer J., Oppegård C., Rogne P., Haugen H.S., Kristiansen P.E. (2010). Structure and Mode-of-Action of the Two-Peptide (Class-IIb) Bacteriocins. Probiotics Antimicrob. Proteins.

[19] Anderssen E., Diep D.B., Nes I.F., Eijsink V.G.H., Nissen-Meyer J. (1998). Antagonistic Activity of Lactobacillus plantarum C11: Two New Two-Peptide Bacteriocins, Plantaricins EF and JK, and the Induction Factor Plantaricin A. Appl. Environ. Microbiol..

[20] Tiwari S.K., Srivastava S. (2008). Purification and characterization of plantaricin LR14: A novel bacteriocin produced by Lactobacillus plantarum LR/14. Appl. Microbiol. Biotechnol..

[21] Cao J., De La Fuente-Nunez C., Ou R.W., Torres M.D.T., Pande S.G., Sinskey A.J., Lu T.K. (2018). Yeast-based synthetic biology platform for antimicrobial peptide production. ACS Synth. Biol..

[22] Perez-Pinera P., Han H., Cleto S., Cao J., Purcell O., Shah K., Lee K., Ram R., Lu T.K. (2016). Synthetic biology and microbioreactor platforms for programmable production of biologics at the point-of-care. Nat. Commun..

[23] Cereghino J.L., Cregg J.M. (2000). Heterologous protein expression in the methylotrophic yeast Pichia pastoris. FEMS Microbiol. Rev..

[24] Sreekrishna K., Brankamp R.G., Kropp K.E., Blankenship D.T., Tsay J.-T., Smith P.L., Wierschke J.D., Subramaniam A., Birkenberger L.A. (1997). Strategies for optimal synthesis and secretion of heterologous proteins in the methylotrophic yeast *Pichia pastoris*. Gene.

[25] Jin F.L., Xu X.-X., Yu X.-Q., Ren S.-X. (2009). Expression and characterization of antimicrobial peptide CecropinAD in the methylotrophic yeast Pichia pastoris. Process Biochem..

[26] Chen Y.Q., Zhang S., Li B.C., Qiu W., Jiao B., Zhang J., Diao Z.Y. (2008). Expression of a cytotoxic cationic antibacterial peptide in Escherichia coli using two fusion partners. Protein Expr. Purif..

[27] Li Y. (2011). Recombinant production of antimicrobial peptides in Escherichia coli: A review. Protein Expr. Purif..

[28] Ishida H., Nguyen L.T., Gopal R., Aizawa T., Vogel H.J. (2016). Overexpression of Antimicrobial, Anticancer, and Transmembrane Peptides in Escherichia coli through a Calmodulin-Peptide Fusion System. J. Am. Chem. Soc..

[29] Yang Y.H., Zheng G., Li G., Zhang X.-J., Cao Z.-Y., Rao Q., Wu K.-F. (2004). Expression of bioactive recombinant GSLL-39, a variant of human antimicrobial peptide LL-37, in *Escherichia coli*. Protein Expr. Purif..

[30] Moon J.Y., Henzler-Wildman K.A., Ramamoorthy A. (2006). Expression and purification of a recombinant LL-37 from *Escherichia coli*. Biochim. Biophys. Acta.

[31] Xu X., Jin F., Yu X., Ren S., Hu J., Zhang W. (2007). High-level expression of the recombinant hybrid peptide cecropinA(1-8)-magainin2(1-12) with an ubiquitin fusion partner in *Escherichia coli*. Protein Expr. Purif..

[32] Tingting T., Wu D., Li W., Zheng X., Fu A., Shan A. (2017). High Specific Selectivity and Membrane-Active Mechanism of Synthetic Cationic Hybrid Antimicrobial Peptides Based on the Peptide FV7. Int. J. Mol. Sci..

[33] Moll G.N., Akker E.V.D., Hauge H.H., Nissen-Meyer J., Nes I.F., Konings W.N., Driessen A.J.M. (1999). Complementary and Overlapping Selectivity of the Two-Peptide Bacteriocins Plantaricin EF and JK. J. Bacteriol..

[34] Zhang X., Wang Y., Liu L., Wei Y., Shang N., Zhang X., Li P. (2016). Two-peptide bacteriocin PlnEF causes cell membrane damage to Lactobacillus plantarum. Biochim. Biophys. Acta.

[35] Ekblad B., Kyriakou P.K., Oppegård C., Nissen-Meyer J., Kaznessis Y.N., Kristiansen P.E. (2016). Structure-Function Analysis of the Two-Peptide Bacteriocin Plantaricin EF. Biochemistry.

[36] Cytryńska M.G., Zdybicka-Barabas A. (2015). Defense peptides: Recent developments. Biomol. Concepts.

[37] Schagger H. (2006). Tricine-SDS-PAGE. Nat. Protoc..

[38] Wiegand I., Hilpert K., Hancock R.E. (2008). Agar and broth dilution methods to determine the minimal inhibitory concentration (MIC) of antimicrobial substances. Nat. Protoc..

[39] Lee J.-K., Seo C.H., Luchian T., Park Y. (2015). Antimicrobial Peptide CMA3 Derived from the CA-MA Hybrid Peptide: Antibacterial and Anti-inflammatory Activities with Low Cytotoxicity and Mechanism of Action in *Escherichia coli*. Antimicrob. Agents Chemother..

