# Supplementary figures and images for: Expression of Hybrid Peptide EF-1 in Pichia pastoris, Its Purification, and Antimicrobial Characterization
Source: Molecules. 2020 Nov 26;25(23):5538. doi: 10.3390/molecules25235538 (PMC7728367; doi:10.3390/molecules25235538)

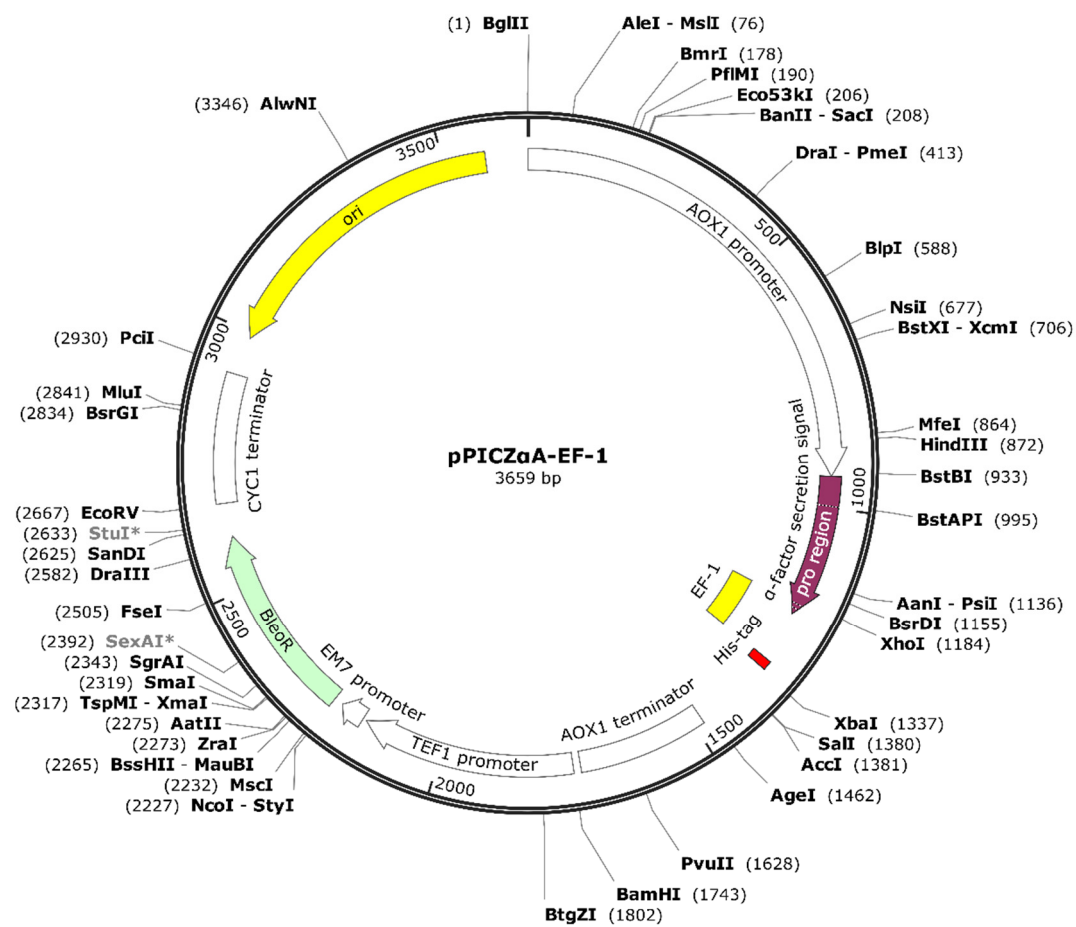

**Supplementary Figure 1.** The map of expression vector construction.

Supplement: Supplementary file 1 [file molecules-25-05538-s001.pdf]
